# A dual‐channel fluorescent probe for in situ imaging of mitochondrial superoxide and microenvironmental changes in Cd‐induced hepatorenal dysfunction

**DOI:** 10.1002/smo2.70105

**Published:** 2026-10-05

**Authors:** Zibo Zhai, Liang Tao, Jia Huang, Xijian Wang, Hailin Zhang, Yuandi Wang, Jiaoli Zhang, Dan Cheng, Longwei He, Songjiao Li

**Affiliations:** ^1^ School of Pharmaceutical Science Hengyang Medical School University of South China Hengyang China; ^2^ Department of Ultrasound Medicine The First Affiliated Hospital Hengyang Medical School University of South China Hengyang China; ^3^ Department of Gastroenterology The Affiliated Nanhua Hospital Hengyang Medical School Clinical Research Institute University of South China Hengyang China

**Keywords:** cadmium, dual‐channel fluorescent probe, ferroptosis, hepatorenal injury, superoxide, viscosity/polarity

## Abstract

Cadmium (Cd) is an environmental pollutant with high bioaccumulation and multi‐organ toxicity, posing a major threat to human health, especially to the liver and kidneys. Fluorescent probes have shown great potential for disease‐related imaging because of their high sensitivity and specificity. However, most existing probes cannot simultaneously image the liver and kidneys in vivo, which limits their application in hepatorenal disease studies. Increasing evidence suggests that ferroptosis, accompanied by mitochondrial dysfunction and oxidative stress, is involved in Cd‐induced hepatorenal injury. Nevertheless, the dynamic changes in key mitochondrial parameters, including reactive oxygen species, polarity, and viscosity, remain poorly understood. Herein, we developed **VPS**, a mitochondria‐targeted dual‐NIR fluorescent probe responsive to mitochondrial superoxide (O_2_^•−^) and microenvironment‐related viscosity/polarity changes. Based on twisted intramolecular charge transfer and ICT mechanisms, VPS showed high sensitivity, good selectivity, and efficient mitochondrial targeting. **VPS** was successfully applied to fluorescence imaging in Cd‐exposed living cells, zebrafish, and mice, revealing signal changes in the liver and kidneys consistent with increased oxidative stress and altered mitochondrial microenvironments. **VPS** also enabled evaluation of the protective effect of N‐acetylcysteine. This probe provides a useful platform for dual‐channel imaging of mitochondrial oxidative stress and microenvironment‐related changes in Cd‐induced hepatorenal injury.

## Graphical abstract

A multi‐analyte‐responsive, dual‐emission fluorescent probe enables simultaneous imaging of viscosity/polarity and superoxide in the liver and kidneys. It reveals CdCl_2_‐induced hepatorenal ferroptosis through oxidative stress, mitochondrial dysfunction, and lipid peroxidation, providing a versatile platform for multi‐organ disease visualization.

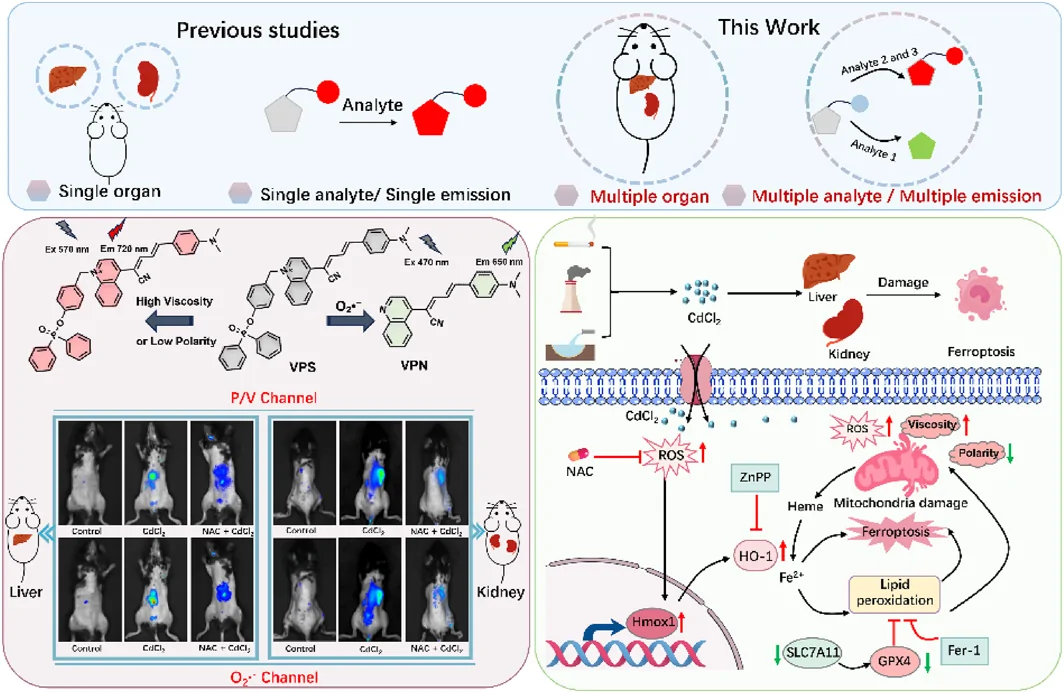

## 1 INTRODUCTION

Cadmium (Cd) is a widespread environmental and occupational toxicant that accumulates in vivo and causes severe multi‐organ damage, particularly in the liver and kidneys[1, 2, 3, 4]. However, current clinical assessment of Cd‐induced hepatorenal injury still relies mainly on serological markers, conventional imaging, and biopsy. Traditional biomarkers such as alanine aminotransferase (ALT), aspartate aminotransferase (AST), blood urea nitrogen (BUN), and creatinine lack sufficient sensitivity and specificity for early‐stage injury[5], while magnetic resonance imaging, ultrasound, and computed tomography are limited by sensitivity, accuracy, or temporal resolution[6, 7]. Although biopsy remains the diagnostic gold standard, its invasiveness and susceptibility to sampling bias limit its practical utility[8]. Therefore, there is a strong need for a sensitive and noninvasive strategy that can dynamically visualize Cd‐induced liver and kidney injury in situ.

Growing evidence suggests that ferroptosis is closely involved in Cd‐induced hepatorenal damage[9]. As an iron‐dependent form of regulated cell death, ferroptosis is characterized by mitochondrial dysfunction, oxidative stress, and lipid peroxidation[10]. Cd exposure disrupts mitochondrial homeostasis, promotes reactive oxygen species (ROS) overproduction, depletes glutathione, and thereby accelerates ferroptotic injury[11]. Among mitochondrial ROS, O_2_^•−^ is an important upstream species associated with mitochondrial oxidative stress and may serve as an informative marker of toxin‐induced organ injury[12]. At the same time, mitochondrial microenvironmental parameters, especially viscosity and polarity, are also closely associated with metabolic dysfunction and disease progression[13]. Because Cd‐induced injury involves not only oxidative stress but also coordinated microenvironmental remodeling, simultaneous monitoring of O_2_^•−^ and viscosity/polarity should provide a more comprehensive assessment than single‐analyte detection.

Fluorescence imaging, owing to its high sensitivity, selectivity, simple operation, noninvasiveness, and real‐time in situ visualization capability, has become an important tool for monitoring bioactive molecules and pathological processes [14, 15]. In Cd‐induced liver and kidney injury, it can dynamically capture oxidative stress‐related signals and mitochondrial microenvironmental changes. Previous studies indicate that mitochondrial O_2_^•−^, polarity, and viscosity are closely linked to ferroptosis: O_2_^•−^ reflects early oxidative stress, whereas polarity and viscosity changes indicate mitochondrial microenvironmental remodeling. Therefore, simultaneous monitoring of mitochondrial O_2_^•−^ and viscosity/polarity‐related signals can more comprehensively reveal oxidative stress‐ and ferroptosis‐associated changes than single‐parameter detection. Developing a fluorescent probe capable of synchronous liver and kidney imaging of these signals is thus important for mechanistic studies and in vivo injury assessment. However, the existing probes still have clear limitations, as summarized in Supporting Information S1: Table S1. First, many probes for liver‐ or kidney‐related diseases respond to only one parameter, such as O_2_^•−^, polarity, or viscosity [16, 17, 18, 19, 20, 21], which is insufficient for multidimensional hepatorenal injury involving elevated ROS, decreased polarity, and increased viscosity. Although useful for specific indicators, such probes cannot fully reveal or compare the real‐time interplay among multiple signals because of differences in concentration, absorption, permeability, and technical constraints. Second, several probes have been applied to hepatorenal injury imaging, but most still rely on a single response signal, limiting their ability to reflect coordinated changes during multi‐organ injury [22, 23, 24, 25, 26]. Third, although multiparameter probes improve multiplexed output and diagnostic reliability [27, 28, 29, 30, 31, 32, 33, 34], their applications are mainly limited to cells, superficial tumors, or single‐organ models, and their signal‐to‐background ratios in deep tissues remain suboptimal. Recent advances in dual‐color organelle imaging, renal‐clearable NIR probes, and dual‐channel sensing further demonstrate the utility of fluorescence imaging for monitoring oxidative stress, microenvironmental changes, and organ dysfunction [35, 36, 37, 38]. Thus, probes capable of synchronously imaging mitochondrial oxidative stress and microenvironmental alterations in both liver and kidney remain limited. Compared with conventional single‐organ or single‐parameter imaging, a mitochondria‐targeted dual‐channel strategy reporting mitochondrial O_2_^•−^ and integrated viscosity/polarity‐associated microenvironmental signals may provide a more comprehensive approach for evaluating Cd‐induced mitochondrial injury and related hepatorenal dysfunction (Figure 1).

**FIGURE 1:**
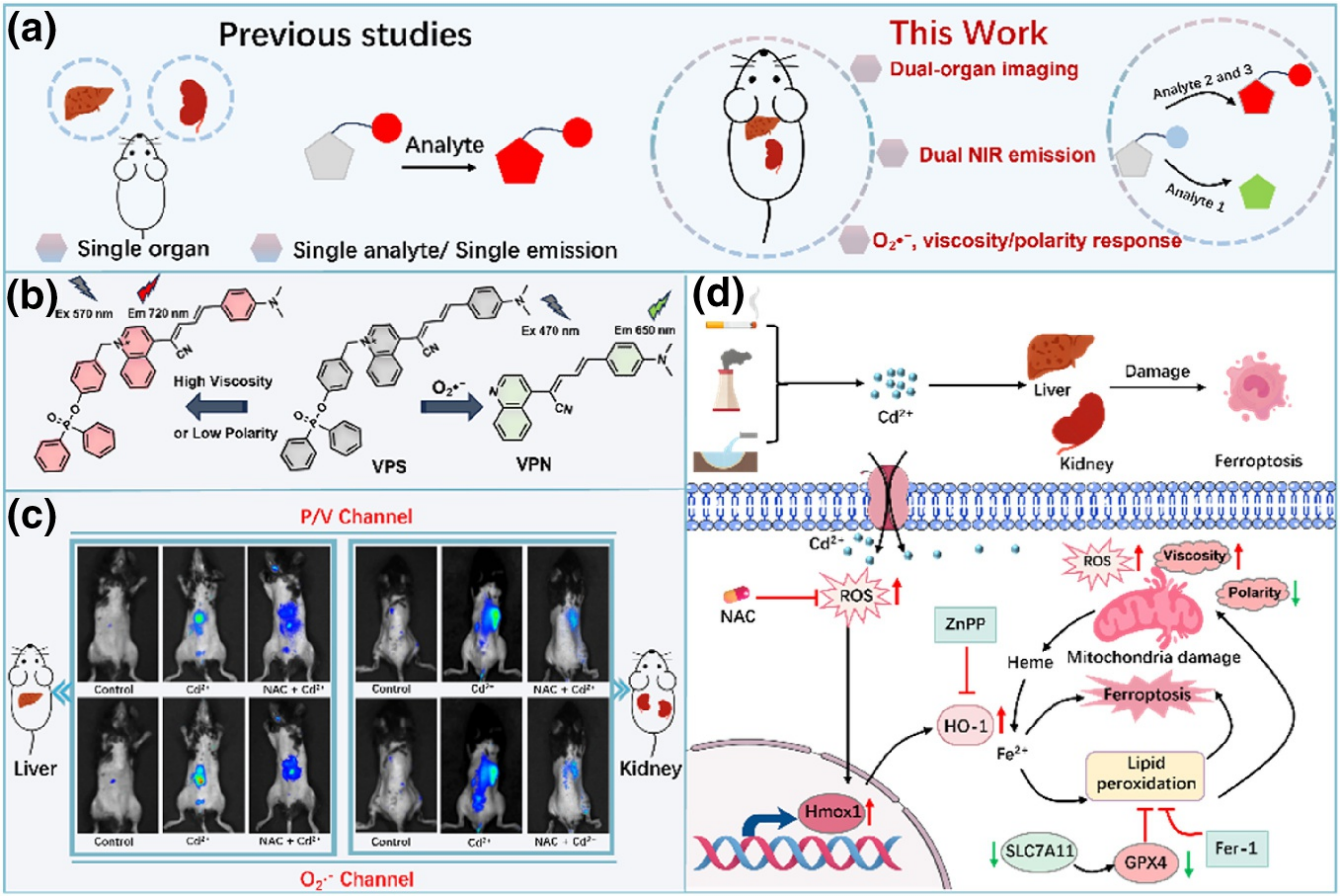
(a) Comparison of multiplexed imaging and traditional imaging. (b) Molecular structure and sensing mechanism of **VPS**. (c) Representative in vivo fluorescence images of the liver and kidney in CdCl_2_‐induced injury mice after **VPS** administration. (d) Schematic illustration of CdCl_2_‐induced liver and kidney injury associated with ferroptotic processes and the protective effect of N‐acetylcysteine pretreatment.

Here, we report **VPS**, a mitochondria‐targeted dual‐channel fluorescent probe for monitoring mitochondrial O_2_^•−^ and integrated viscosity/polarity‐related microenvironmental changes during Cd‐induced liver and kidney injury. **VPS** was rationally designed based on twisted intramolecular charge transfer and intramolecular charge transfer mechanisms, with the quinolinium unit enabling mitochondrial targeting. It responds to O_2_^•−^ at 650 nm and to viscosity/polarity‐associated changes at 740 nm, allowing dual near‐infrared imaging of oxidative stress and mitochondrial microenvironmental remodeling. In live cells, zebrafish, and mice, **VPS** effectively tracked these pathological signals and enabled synchronous in vivo visualization of Cd‐induced hepatorenal injury, revealing excessive mitochondrial O_2_^•−^ generation together with increased viscosity and/or decreased polarity. This dual‐channel mitochondrial imaging strategy provides a more comprehensive approach than conventional single‐organ or single‐parameter methods for assessing oxidative stress and microenvironmental alterations during Cd‐induced ferroptosis‐related hepatorenal injury. **VPS** also enabled fluorescence‐based evaluation of the protective effect of N‐acetylcysteine (NAC) pretreatment, which biochemical and molecular analyses suggested may be associated with reduced ferroptosis‐related oxidative stress and HO‐1‐related changes. Overall, **VPS** is a useful tool for in situ monitoring of mitochondrial oxidative stress and microenvironmental remodeling and shows promise for mechanistic studies and protective‐intervention evaluation in Cd‐induced liver and kidney injury.

## 2 EXPERIMENTAL SECTION

Materials, probe synthesis, characterization, cell culture, and animal experiments are fully described in the Supporting Information. The structural characterization and purity assessment of the synthesized compounds, including ^1^H/^13^C NMR, HRMS, and HPLC analyses, are provided in the Supporting Information (Supporting Information S1: Figures S1–S8).

## 3 RESULTS AND DISCUSSION

### 3.1 Optical responses of **VPS**

The polarity sensitivity of **VPS** was assessed by recording its absorption and fluorescence spectra in solvents with different polarities. As shown in Supporting Information S1: Figure S9, the absorption spectra changed negligibly, indicating that the ground‐state dipole moment of **VPS** was minimally affected by solvent polarity. In contrast, fluorescence spectra showed clear polarity dependence, lower solvent polarity enhanced fluorescence intensity and induced a blue‐shifted emission (Supporting Information S1: Figure S10). In 1,4‐dioxane/water mixtures, increasing the dioxane fraction from 0% to 90% progressively decreased solvent polarity and markedly increased **VPS** fluorescence (Figure 2a). *F*_max_ showed a strong linear correlation with the polarity parameter *Δf* (Figure 2b), confirming **VPS** as a polarity‐sensitive probe. **VPS** also showed minimal pH sensitivity (Supporting Information S1: Figure S11), supporting its stability and reliability for polarity detection.

The viscosity‐responsive behavior of **VPS** was evaluated first. As shown in Supporting Information S1: Figure S12, **VPS** showed viscosity‐dependent absorption changes with higher absorbance around 600 nm in glycerol than in water. Fluorescence measurements showed negligible emission in water but strong emission at 740 nm in glycerol (Supporting Information S1: Figure S13). In glycerol/water mixtures, I_740_ gradually increased as the glycerol fraction rose from 0% to 90% (Figure 2c), likely because high viscosity restricted intramolecular rotation, promoting charge transfer and radiative transition. LogI_740_ showed a strong linear relationship with log *η* (*R* ^2^ = 0.9924; Figure 2d). Similar viscosity‐dependent behavior was further confirmed in the methanol–glycerol mixtures (Supporting Information S1: Figure S14). **VPS** also exhibited stable fluorescence in water/glycerol (1:9, v/v) over pH 3–11 (Supporting Information S1: Figure S15), confirming its stability and sensitivity as a viscosity‐responsive probe. The fluorescence quantum yield of VPS was further evaluated and increased from 0.032 to 0.16 and 0.13 under polarity‐ and viscosity‐responsive conditions, respectively.

The reactivity of **VPS** toward O_2_^•−^ was then examined. After O_2_^•−^ addition, the absorption peak at 600 nm decreased markedly, accompanied by a new band at 430 nm (Supporting Information S1: Figure S16), indicating a specific reaction. Meanwhile, fluorescence at 650 nm was strongly enhanced (Supporting Information S1: Figure S17). Under 480 nm excitation, the background signal was negligible, while I_650_ increased with O_2_^•−^ concentration from 0 to 80 μM (Figure 2e), giving a detection limit of 56 nM by the 3σ/k method (Figure 2f). **VPS** showed a stable O_2_^•−^ response over pH 3–11, with optimal performance near physiological pH (Supporting Information S1: Figure S18), and reached saturation within 10 min (Supporting Information S1: Figure S19). Selectivity and anti‐interference assays confirmed that only O_2_^•−^ induced significant fluorescence enhancement at 650 nm, even with competing species (Supporting Information S1: Figures S20 and S21). HRMS revealed a peak at m/z 326.1668, matching VPN [M+H]^+^ (calcd m/z 326.1661), confirming product formation (Supporting Information S1: Figure S22). DFT calculations at the B3LYP/6‐31G(d,p) level showed HOMO–LUMO gaps of 1.93 eV for **VPS** and 3.05 eV for VPN, supporting the observed blue‐shifted emission after reaction with O_2_^•−^ (Supporting Information S1: Figure S23). These results support the proposed conversion of **VPS** to VPN by O_2_^•−^ (Figure 1b).

**FIGURE 2:**
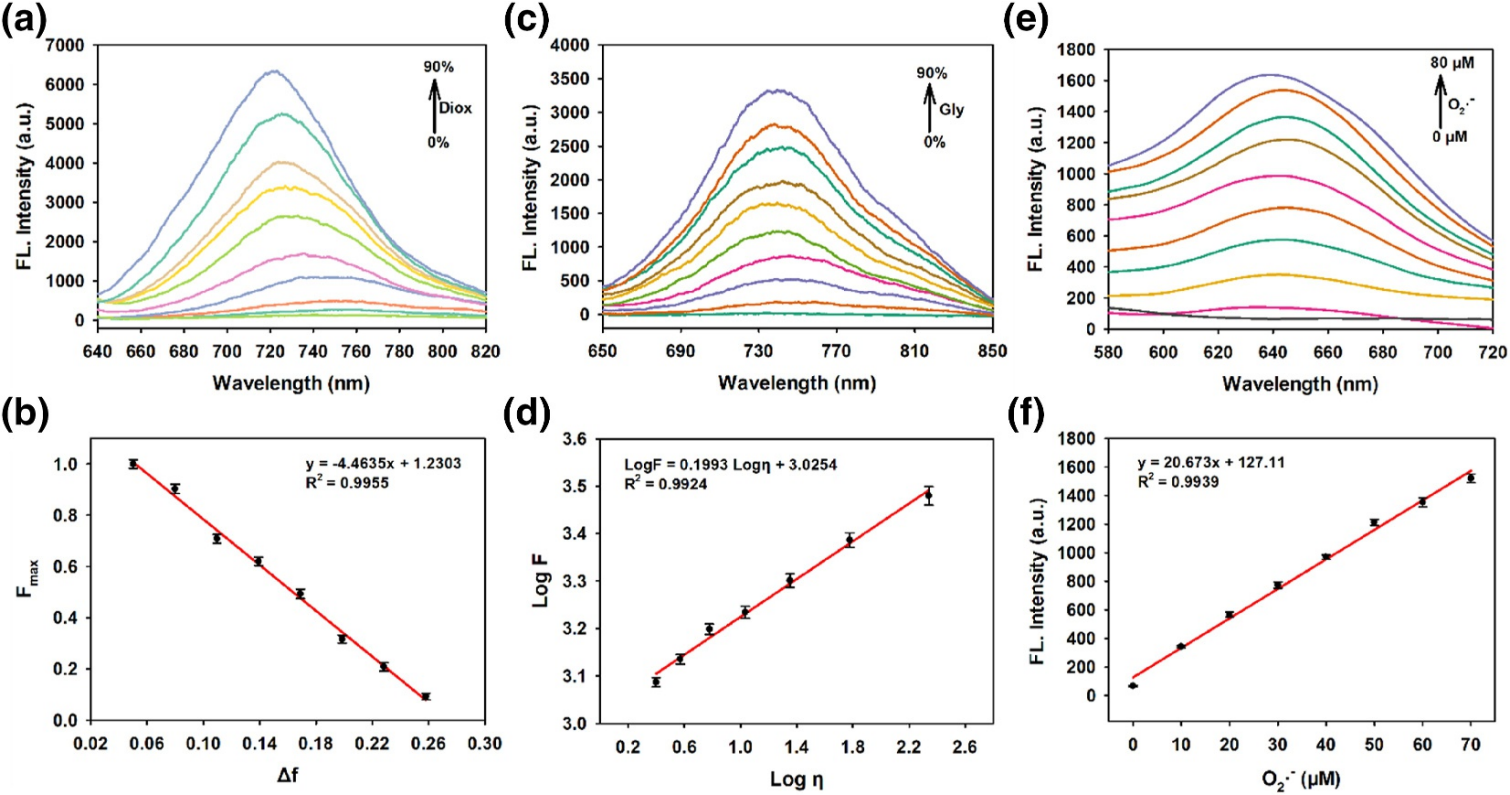
(a) Fluorescence spectra of **VPS** in 1,4‐dioxane/H_2_O mixtures with varying dioxane fractions. λ_ex_/λ_em_ = 600/740 nm. (b) Linear relationship between *F*_max_ and solvent polarity Δ*f*. (c) Fluorescence spectra of **VPS** (10 μM) with increasing viscosity induced by glycerol fractions from 0% to 90%. λ_ex_/λ_em_ = 600/740 nm. (d) Plot of Log Fmax versus log η. (e) Fluorescence spectra of **VPS** (10 μM) with increasing O_2_^•−^ concentrations. (f) Linear relationship between **VPS** fluorescence intensity and O_2_^•−^ concentration. λ_ex_/λ_em_ = 480/650 nm. Data are presented as mean ± SD (*n* = 3).

Spectral crosstalk between the response channels was further assessed. Under 480 nm excitation, O_2_^•−^ caused a pronounced fluorescence increase, whereas 50% glycerol, 50% dioxane, and their mixture induced only weak to moderate changes (Supporting Information S1: Figure S24A). Conversely, under 600 nm excitation, O_2_^•−^ produced little fluorescence variation, while glycerol, dioxane, and their mixture enhanced emission, with the mixed system giving the strongest signal (Supporting Information S1: Figure S24B). Thus, the O_2_^•−^ channel is mainly activated at 480 nm, whereas the viscosity/polarity channel is read out at 600 nm with minimal mutual interference. Increased viscosity and decreased polarity both enhanced red‐channel emission, and their coexistence further amplified the signal. Since liver and kidney injury are commonly associated with increased viscosity, decreased polarity, and elevated ROS levels [16, 17, 18, 19, 20, 21], **VPS** may sensitively report overall microenvironmental abnormalities through dual‐amplified responses. The viscosity‐ and polarity‐dependent fluorescence responses of VPN were further examined. Negligible fluorescence changes were observed under different viscosity and polarity conditions (Supporting Information S1: Figure S25), indicating that VPN is essentially insensitive to these microenvironmental parameters.

### 3.2 Cytotoxicity and co‐localization experiments

Before intracellular experiments, the cytotoxicity of **VPS** was evaluated by MTT assay in HepG2 and HK‐2 cells. After incubation with 0–20 μM **VPS** for 12 or 24 h, both cell lines maintained >80% viability, indicating good biocompatibility (Supporting Information S1: Figure S26). The subcellular localization of **VPS** was then examined by co‐localization imaging with Mito Tracker Green in living cells. **VPS** showed strong overlap with the mitochondrial marker with Pearson correlation coefficients of 0.98 in HepG2 cells and 0.96 in HK‐2 cells, confirming its excellent mitochondrial targeting ability. In contrast, **VPS** showed limited co‐localization with lysosomes and lipid droplets with PCC values of 0.39 and 0.41 in HepG2 cells and 0.38 and 0.32 in HK‐2 cells, respectively (Supporting Information S1: Figure S27), further supporting its preferential mitochondrial localization.

### 3.3 Imaging of O_2_^•−^, viscosity/polarity in living cells

We next assessed whether **VPS** could monitor mitochondrial polarity and viscosity changes in living cells. Because the viscosity‐ and polarity‐related emissions partially overlap, both signals were collected in the red channel. Therefore, their individual contributions cannot be quantitatively distinguished in biological systems, and the red‐channel fluorescence is interpreted as an integrated microenvironmental signal reflecting increased viscosity and/or decreased polarity. CCCP, a mitochondrial uncoupler that dissipates the proton gradient and reduces membrane potential, was used to induce polarity reduction. Increasing CCCP concentrations markedly enhanced red‐channel fluorescence in both cell lines (Supporting Information S1: Figures S28A,C and S29A,C), demonstrating that **VPS** effectively responds to mitochondrial polarity changes.

To further evaluate the viscosity‐sensing ability of **VPS**, HepG2 and HK‐2 cells were treated with nystatin (Nys) or monensin (Mon), both of which increase mitochondrial viscosity. **VPS** fluorescence was significantly enhanced in a concentration‐dependent manner after Nys or Mon treatment (Supporting Information S1: Figures S28B,D and S29B,D), confirming its ability to detect mitochondrial viscosity changes in living cells.

The capacity of **VPS** to image intracellular O_2_^•−^ was then assessed. Phorbol 12‐myristate 13‐acetate (PMA), an endogenous O_2_^•−^/ROS inducer, caused concentration‐dependent fluorescence enhancement in HepG2 and HK‐2 cells (Supporting Information S1: Figures S28E,F and S29E,F). This response was markedly inhibited by the O_2_^•−^ scavengers TEMPO and Tiron, confirming the selective response of **VPS** toward O_2_^•−^. N‐acetylcysteine treatment after PMA stimulation also significantly reduced green fluorescence, demonstrating that **VPS** enables real‐time imaging of intracellular O_2_^•−^ fluctuations.

Because inflammation is closely associated with liver and kidney injury, an LPS‐stimulated inflammatory cell model was established. Increasing LPS concentrations gradually enhanced both green O_2_^•−^‐related and red viscosity/polarity‐related signals, indicating elevated oxidative stress and mitochondrial microenvironmental alterations (Figure 3a–c and S30A–S30C). N‐acetylcysteine treatment significantly reduced both signals, supporting the use of **VPS** for monitoring inflammation‐associated cellular changes.

Finally, **VPS** was applied to ferroptosis‐related injury models. Erastin (Era), a typical ferroptosis inducer, markedly increased both green and red fluorescence, whereas ferrostatin‐1 (Fer‐1) substantially suppressed these responses (Figure 3d–f and Supporting Information S1: Figure S30D–F). These results indicate that **VPS** can report O_2_^•−^ elevation and mitochondrial microenvironmental disturbance under ferroptosis‐related conditions, with the red‐channel signal reflecting increased viscosity and/or decreased polarity. Notably, similar O_2_^•−^ elevation and mitochondrial microenvironmental alterations were also observed in LPS‐induced inflammation and erastin‐induced ferroptosis models, suggesting that these changes may not be unique to CdCl_2_‐induced injury.

**FIGURE 3:**
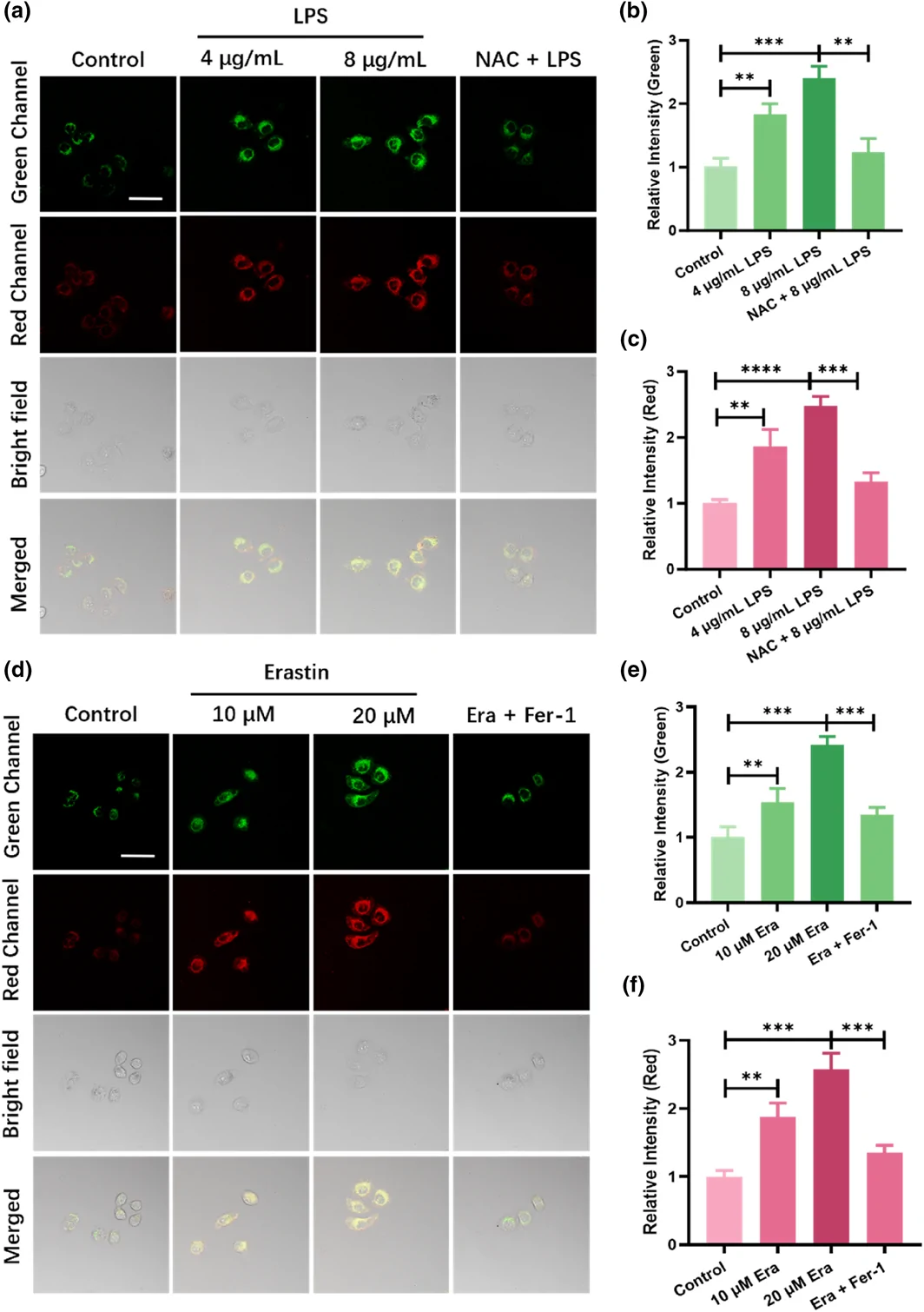
(a) Fluorescence images of VPS‐stained HepG2 cells treated with LPS (4 or 8 μg/mL, 12 h) or LPS (8 μg/mL, 12 h) plus N‐acetylcysteine (NAC) (500 μM, 1 h). (b), (c) Quantification of O_2_^•−^ and integrated viscosity/polarity‐associated fluorescence signals from (a). (d) Fluorescence images of VPS‐stained HepG2 cells treated with Era (10 or 20 μM, 12 h) or Era (20 μM, 12 h) plus Fer‐1 (10 μM, 12 h). (e, f) Quantification of O_2_^•−^ and integrated viscosity/polarity‐associated fluorescence signals from (d). VPS: 10 μM. Scale bar: 20 μm.

### 3.4 Simultaneous tracking of O_2_^•−^ and microenvironment‐related changes in CdCl_2_‐injured cells and zebrafish

CdCl_2_ is widely distributed in modern ecosystems and poses serious environmental and health risks. To assess whether **VPS** could monitor O_2_^•−^‐ and viscosity/polarity‐related fluorescence changes in CdCl_2_‐injured cells, HepG2 and HK‐2 cells were used as models. Cells incubated with **VPS** alone showed weak red viscosity/polarity and green O_2_^•−^ fluorescence, whereas CdCl_2_ pretreatment (10 or 20 μM, 24 h) induced concentration‐dependent enhancement in both channels (), indicating increased O_2_^•−^ levels and mitochondrial microenvironmental perturbation, including increased viscosity and/or decreased polarity. N‐acetylcysteine markedly attenuated these fluorescence increases and restored the signals toward control levels, supporting its protection against CdCl_2_‐induced oxidative injury. Compared with single‐parameter probes, VPS simultaneously reports mitochondrial O_2_^•−^‐related oxidative stress and viscosity/polarity‐associated microenvironmental changes. This synchronous dual‐channel strategy enables direct comparison of these complementary signals and provides a more comprehensive view of Cd‐induced mitochondrial dysfunction than either signal alone.

To examine the relationship between fluorescence changes and ferroptosis‐related processes, cells were pretreated with ferrostatin‐1 (Fer‐1), a ferroptosis inhibitor, or zinc protoporphyrin IX (ZnPP), a competitive HO‐1 inhibitor, before **VPS** incubation. Both Fer‐1 and ZnPP significantly suppressed CdCl_2_‐induced fluorescence enhancement in the O_2_^•−^ and viscosity/polarity channels (Figure 4a–f). Flow cytometry further confirmed reduced CdCl_2_‐triggered increases in both channels in HepG2 and HK‐2 cells after Fer‐1 or ZnPP treatment (Supporting Information S1: Figure S31A–D). Fer‐1 may attenuate the fluorescence responses by suppressing ferroptosis‐associated lipid peroxidation, whereas ZnPP may reduce HO‐1‐associated oxidative/iron‐dependent stress, thereby alleviating O_2_^•−^ accumulation and mitochondrial microenvironmental disturbance. These inhibitor experiments further support the association of the CdCl_2_‐induced dual‐channel responses with ferroptosis‐related processes and HO‐1‐associated oxidative stress.

**FIGURE 4:**
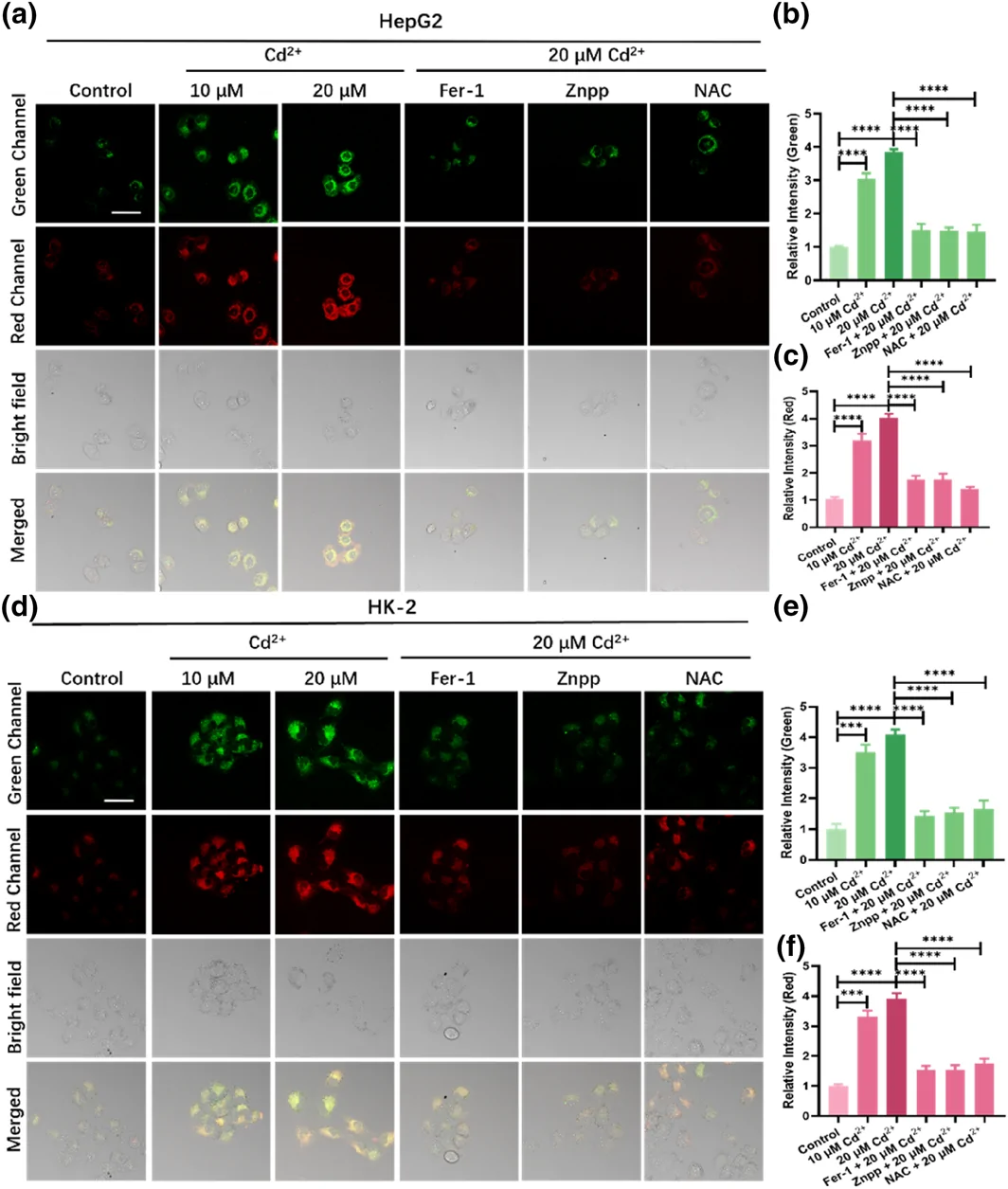
CdCl_2_‐induced oxidative stress model. Fluorescence imaging of VPS in HepG2 cells (a) and HK‐2 cells (d). Cells were treated with VPS (10 μM, 30 min) alone (control), with CdCl_2_ (10 or 20 μM, 24 h) followed by VPS, or pretreated with CdCl_2_ (20 μM, 24 h) plus Fer‐1 (10 μM, 6 h), ZnPP (10 μM, 6 h), or N‐acetylcysteine (500 μM, 1 h) followed by VPS. Quantification of fluorescence intensities in the O_2_^•−^ and viscosity/polarity channels from HepG2 images is shown in (b, c), and from HK‐2 images in (e, f). Scale bar: 20 μm.

Transcriptomic analysis further supported these findings. CdCl_2_ induced broad transcriptional changes in kidney tissue, with KEGG enrichment highlighting ferroptosis‐related pathways (Supporting Information S1: Figure S32A,B), and volcano plot analysis showed marked Hmox1 upregulation (Supporting Information S1: Figure S32C). Similarly, liver transcriptomic analysis (GSE302882) revealed distinct gene expression changes and ferroptosis‐related pathway enrichment after CdCl_2_ treatment (Supporting Information S1: Figure S32D,E). CdCl_2_ also significantly increased Hmox1 mRNA levels in both kidney and liver tissues (Supporting Information S1: Figure S32F,G). These transcriptomic data further support the involvement of ferroptosis‐associated pathways and Hmox1 upregulation in CdCl_2_‐induced hepatorenal injury.

To further assess these alterations at the cellular level, Hmox1 expression was examined in HK‐2 and HepG2 cells. CdCl_2_ significantly increased Hmox1 mRNA levels in both cell lines, consistent with the in vivo transcriptomic results (Supporting Information S1: Figure S33A,B). Western blotting showed that CdCl_2_ markedly downregulated the ferroptosis‐related proteins GPX4 and SLC7A11, while upregulating the oxidative stress‐responsive protein HMOX1 (Supporting Information S1: Figure S33C,F). These changes were partially reversed by Fer‐1, ZnPP, or NAC in both cell lines. These molecular changes were consistent with the imaging and transcriptomic evidence of ferroptosis‐related oxidative injury.

Zebrafish larvae were further used to evaluate whether **VPS** could report O_2_^•−^‐ and viscosity/polarity‐related fluorescence changes in vivo. **VPS**‐treated larvae showed weak green O_2_^•−^‐channel and red viscosity/polarity‐channel fluorescence, whereas CdCl_2_ pretreatment (10 or 20 μM, 24 h) induced concentration‐dependent enhancement in both channels (Supporting Information S1: Figure S34A,C). These changes indicate CdCl_2_‐induced O_2_^•−^ elevation and mitochondrial microenvironmental perturbation, including increased viscosity and/or decreased polarity.N‐acetylcysteine pretreatment significantly suppressed the dual‐channel response and restored the signals toward control levels, supporting its protective effect in vivo.

### 3.5 In vivo biocompatibility evaluation of VPS

Before disease‐related applications, the in vivo biocompatibility of **VPS** was evaluated. Major organs and blood samples were collected 24 h after intravenous **VPS** injection. H&E staining revealed no obvious pathological abnormalities, including necrosis, hemorrhage, inflammation, or tissue damage, in the heart, liver, spleen, lungs, or kidneys compared with controls (Supporting Information S1: Figure S35A). Serum ALT, AST, BUN, and CRE levels showed no significant differences between **VPS**‐treated and control mice, indicating no detectable hepatotoxicity or renal dysfunction (Supporting Information S1: Figure S35B,C). Blood compatibility was assessed by hemolysis assay, and diluted RBC suspensions incubated with different **VPS** concentrations showed no obvious hemolysis, with hemolysis ratios below 5% (Supporting Information S1: Figure S35D,E). These results demonstrate the good in vivo biocompatibility and biosafety of **VPS**, supporting its use in subsequent fluorescence imaging studies.

### 3.6 In vivo fluorescence imaging of CdCl_2_‐induced hepatorenal injury

**VPS** was further applied for dual‐channel in vivo fluorescence imaging of CdCl_2_‐induced hepatorenal injury in mice. As shown in Figure 5a, the injury model was established by daily intraperitoneal CdCl_2_ administration for 7 consecutive days, and NAC was administered 2 h before CdCl_2_ in the NAC pretreatment group. After tail‐vein **VPS** administration, imaging was performed as scheduled. Serum assays showed that CdCl_2_ significantly elevated ALT, AST, BUN, and CRE levels, confirming liver and kidney dysfunction, whereas NAC pretreatment markedly alleviated these changes (Supporting Information S1: Figure S36A,D).

Renal imaging in the prone position showed weak fluorescence in both the green O_2_^•−^ channel and red viscosity/polarity channel in control mice, but strong kidney‐region fluorescence after CdCl_2_ treatment (). These signals were substantially reduced by NAC pretreatment. Similarly, liver imaging in the supine position showed strong CdCl_2_‐induced dual‐channel fluorescence, which was attenuated by NAC (Figure 5e–g), indicating oxidative stress and mitochondrial microenvironmental perturbations in both organs.

**FIGURE 5:**
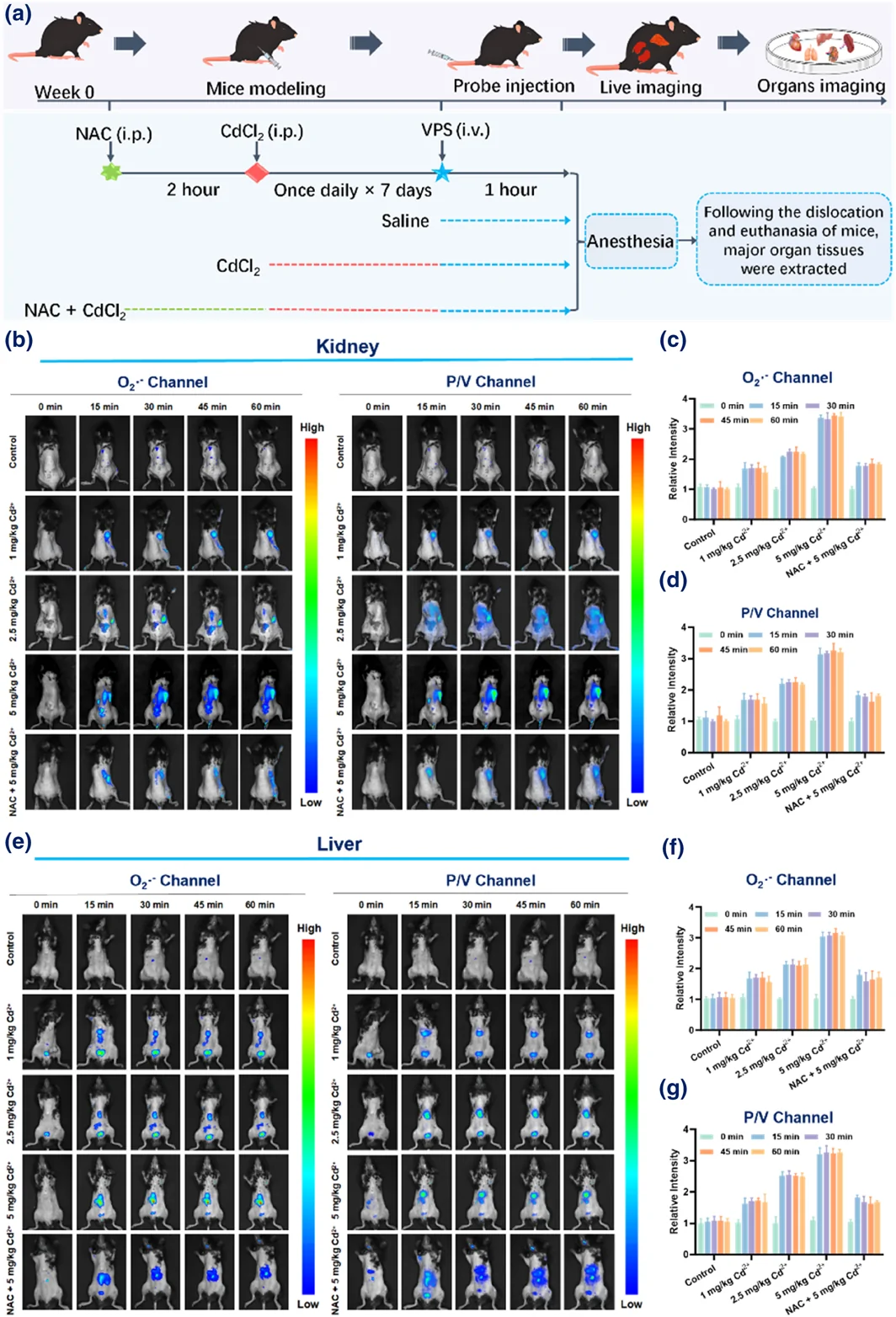
Fluorescence imaging in a CdCl_2_‐induced mouse model. (a) Schematic of the CdCl_2_ exposure protocol and in vivo imaging timeline. (b) In vivo fluorescence imaging of the kidney region in the O_2_^•−^ and viscosity/polarity channels, with corresponding quantification of O_2_^•−^ (c) and viscosity/polarity signals (d). (e) In vivo fluorescence imaging of the liver region in the O_2_^•−^ and viscosity/polarity channels, with corresponding quantification of O_2_^•−^ (f) and viscosity/polarity signals (g). Data are presented as mean ± SD (*n* = 3 mice per group).

Histological analysis confirmed CdCl_2_‐induced hepatorenal injury (Supporting Information S1: Figure S37). Compared with controls, CdCl_2_‐treated livers showed dose‐dependent inflammatory infiltration, hepatocellular degeneration, and focal necrosis, while kidneys exhibited tubular epithelial degeneration, vacuolation, and structural disruption. N‐acetylcysteine pretreatment markedly mitigated these lesions. CdCl_2_ also reduced body weight gain in a dose‐dependent manner, which was partially restored by NAC (Supporting Information S1: Figure S38).

Ex vivo imaging showed strong fluorescence mainly in the liver and kidneys of CdCl_2_‐treated mice, with weak signals in other organs (Supporting Information S1: Figure S39). Quantification confirmed significant increases in both O_2_^•−^‐ and viscosity/polarity‐related fluorescence after CdCl_2_ exposure, which were suppressed by NAC (Supporting Information S1: Figure S40A–F). Similar trends were observed in tissue sections (Supporting Information S1: Figure S41). Together, the biochemical, histological, ex vivo, and in vivo results demonstrate that **VPS** enables in situ visualization of CdCl_2_‐induced hepatorenal injury and associated oxidative and microenvironmental changes, and can evaluate NAC‐mediated protection against Cd‐induced organ damage.

## 4 CONCLUSIONS

In conclusion, **VPS** was developed as a mitochondria‐targeted dual‐NIR fluorescent probe for monitoring mitochondrial O_2_^•−^ and integrated viscosity/polarity‐associated microenvironmental signals. In living cells, zebrafish, and mice, **VPS** enabled real‐time, noninvasive visualization of fluorescence changes associated with CdCl_2_‐induced oxidative stress and mitochondrial microenvironmental disturbance. Combined with biochemical, histopathological, transcriptomic, and protein‐expression analyses, these results indicate that CdCl_2_‐induced hepatorenal injury involves ferroptosis‐related oxidative stress and HMOX1‐related changes. N‐acetylcysteine pretreatment partially attenuated these alterations, supporting its protective effect. Overall, **VPS** provides a useful in situ imaging tool for evaluating CdCl_2_‐related hepatorenal injury and protective interventions.

## CONFLICT OF INTEREST STATEMENT

The authors declare no conflicts of interest.

## ETHICS STATEMENT

All animal experiments conducted in this study were performed in strict accordance with the guidelines established by the Animal Management and Ethics Committee of the Hengyang Medical School, University of South China, with the approval of Protocol No. 2023‐59.

## Supporting information

Supporting Information S1

## Data Availability

The data that supports the findings of this study are available in the supplementary material of this article.
