# Supplementary material for: A dual‐channel fluorescent probe for in situ imaging of mitochondrial superoxide and microenvironmental changes in Cd‐induced hepatorenal dysfunction
Source: Smart Mol. 2026 Oct 5:e70105. Online ahead of print. doi: 10.1002/smo2.70105 (PMC13639408; doi:10.1002/smo2.70105)
Supplement: Supplementary file 1 [file SMO2-9999-0-s001.docx]

**Supporting Information**

**A dual-channel fluorescent probe for in situ imaging of mitochondrial superoxide and microenvironmental changes in Cd-induced hepatorenal dysfunction**

Zibo Zhai^a1^, Liang Tao^a1^, Jia Huang^b^, Xijian Wang^a^, Hailin Zhang^a^, Yuandi Wang^a^, Jiaoli Zhang^b^, Dan Cheng^a,c^, Longwei He^a,b*^ and Songjiao Li,^a*^

^a^ School of Pharmaceutical Science, Hengyang Medical School, University of South China, Hengyang 421001, China.

^b^ Department of Ultrasound Medicine, The First Affiliated Hospital, Hengyang Medical School, University of South China, Hengyang 421002, China.

^c^ Department of Gastroenterology, Clinical Research Institute, The Affiliated Nanhua Hospital, Hengyang Medical School, University of South China, Hengyang 421002, P.R. China.

^1^ Zibo Zhai and Liang Tao contributed equally to this work.

*Corresponding authors:

helongwei0110@163.com (L. He),

lisongjiaoo@126.com (S. Li).

**Table of contents**

[Experimental Procedures	S3](#_Toc447465955)

Additional Table and Figures	S9

**Experimental section**

**Instruments and materials.**

All chemicals were obtained from commercial suppliers and were used directly in the experiments without treatment, except where indicated. The ^1^H NMR and ^13^C NMR spectra were obtained from Bruker Avance 500 NMR spectrometer (Germany). The pH measurements were conducted with PHS-25 pH meter. The ultrapure water was obtained from BYJ1-10-AD. The UV-vis and fluorescence spectra were measured on Shimazu UV-2600 spectrophotometer and Hitachi F-7100 spectrophotometer, respectively. The mass spectra were acquired from Agilent 1100 LC MSD/TOF mass spectrometer. The cell cytotoxicity was measured with a Biotek 800TS. Cellular imaging was acquired on a NIKON A1.

**Calculation of the limit of detection.**

The limit of detection (LOD) for O₂^•⁻^ was calculated based on the following equation: LOD = 3σ/ k. Where σ represents the standard deviation and k represents the slope of the titration spectra curve among the limited range.

**Spectrophotometric Measurements.**

The stock solution (1.0 mM) of VPS was prepared in DMSO. Other stock solutions (thiols, anions, cations, and amino acids) were prepared in ultrapure water. Other concentrations were prepared from the dilution. The fluorescence spectral experiments were completed in PBS buffer (10 mM, 1% DMSO, pH 7.4). CAUTION: CdCl₂ is highly toxic and should be handled with appropriate personal protective equipment and proper waste-disposal procedures.

**Quantum yield measurements**

The fluorescence quantum yields of **VPS** were determined using the relative method, with Nile Red as the reference dye for both the viscosity- and polarity-responsive conditions. The absorbance values of **VPS** and Nile Red at the corresponding excitation wavelengths were maintained below 0.05 to minimize inner-filter effects.

The quantum yields were calculated by comparing the integrated fluorescence intensities and absorbance values of **VPS** with those of Nile Red according to the following equation:

QY_u_ = QY_s_ (m_u_/m_s_) (n_u_/n_s_)^2^.

Where QY is the fluorescence quantum yield, m is the slope obtained from the corresponding plots, and n is the refractive index. The subscripts “s” and “u” refer to the standard and unknown sample, respectively. A series of concentrations of the standard and sample solutions were measured to obtain the corresponding slopes.

**Cell Culture and Viability Assay.**

HepG2 cells were cultured in Dulbecco’s modified Eagle’s medium (DMEM, high glucose; Thermo Scientific) supplemented with 10% fetal bovine serum (FBS) and 1% penicillin/streptomycin. HK-2 cells were maintained in Dulbecco’s modified Eagle’s medium/Nutrient Mixture F-12 (DMEM/F12, 1:1; Thermo Scientific) containing 10% FBS and 1% penicillin/streptomycin. All cells were incubated at 37 °C in a humidified atmosphere of 5% CO₂ and 95% air. Two days before imaging, the cells were passaged and seeded into glass-bottomed dishes (NEST). For labeling, cells were washed with serum-free medium and then incubated with 10 μM **VPS** (containing 1% DMSO) for 30 min at 37 °C.

**Cytotoxicity assay**

HepG2 or HK-2 cells were seeded in 96-well plates and incubated with different concentrations of **VPS** (0, 2.5, 5, 7.5, 10, 15, and 20 μM; containing 1% DMSO in 200 μL of the corresponding culture medium) for 12 or 24 h at 37 °C in a humidified atmosphere of 5% CO₂ and 95% air. Subsequently, 20 μL of 5.0 mg/mL MTT solution was added to each well, followed by incubation for 4 h under the same conditions. After incubation, 100 μL of supernatant was removed from each well, and 150 μL of DMSO was added. The plates were shaken for 10 min, and the absorbance at 490 nm was measured using a microplate reader. Cell survival rate was calculated as A/A₀ × 100%, where A and A₀ represent the absorbance of the experimental and control groups, respectively.

**Confocal imaging in living cells**

HepG2 or HK-2 cells were incubated in the corresponding culture medium containing 10 μM **VPS** for 30 min at 37 °C with 5% CO₂. The cells were then washed three times with 1×PBS and subsequently stained with 500 nM commercial dyes and 10 μΜ **VPS** for 30 min. Green channel for commercial dyes, λ_ex_ = 488 nm, λ_em_ = 500−550 nm. Red channel for **VPS**, λ_ex_ = 561 nm, λ_em_ = 700−760 nm. Images were processed using ImageJ software.

**Flow cytometry analysis**

Before FACS analysis, cells were treated under the same conditions as those used for fluorescence imaging, then washed with PBS two times. After cell detaching by digestion, cells were centrifuged at 1000 rpm for 5 min, resuspended once with PBS, continued to centrifuge at 1000 rpm for 5 min, and resuspended with 500 μL of PBS then placed on ice for measurement. Cell fluorescence signals were collected using a BDFACS Caliber Flow Cytometer (FACS Calibur, Becton Dickinson (BD) Biosciences, San Jose, CA, USA). Cells that were not treated with inhibitors were used as controls. Three independent experiments were performed and the resulting data were analyzed using the FlowJo.

**Western blotting**

Western blotting was carried out as previously described. HK-2 and HepG2 cells were gently rinsed 2-3 times with pre-cooled phosphate-buffered saline (PBS) to remove residual culture medium. An appropriate volume of pre-cooled RIPA lysis buffer (supplemented with protease inhibitors and phosphatase inhibitors) was added to the culture dish. Cells were carefully scraped off using a cell scraper and collected into pre-cooled EP tubes. The EP tubes containing cell lysates were placed on ice for 30 minutes of lysis, with gentle mixing at intervals. The cell lysates were centrifuged at 4 °C at 12, 000 × g for 15 min, and then the supernatants were harvested, which yielded the cell lysate containing the target protein. Protein samples (10 μL/lane) were separated by electrophoresis on 10% sodium dodecyl sulfate-polyacrylamide gels (ACE Biotechnology, China) and then transferred onto PVDF membranes (LABSELECT, China). The corresponding membranes were cut according to the molecular weight of the protein to be detected. The membranes were blocked with 5% skim milk at room temperature for 1 h and washed three times with TBST. After incubation with the corresponding primary antibody overnight at 4 °C, the membranes were washed three times with TBST, and then incubated with the secondary antibody (Cell Signaling Technology) of the corresponding species for 1h at room temperature. Finally, the membranes were incubated with ECL solution (Biosharp, China) and the signal was detected using the imaging system from (MINICHEMI, China). Image J software program was used for densitometric mea surements of the bands and then normalized to the GAPDH loading control. All above experiments were repeated three times.

The following primary antibodies are used: anti-HMOX1 (1:1,000; AF5393, Affinity Biosciences, China), anti-SLC7A11 (1:1,000; DF12509, Affinity Biosciences, China) and anti-GPX4 (1:1,000; DF6701, Affinity Biosciences, China).

**Mice models of CdCl₂ exposure.**

Male C57BL/6J mice (7 weeks old) were acclimated for 1 week under controlled environmental conditions (temperature 22–25 °C, relative humidity 50%) with a 12 h light/12 h dark cycle prior to experimentation. The mice were then randomly divided into five groups: Control group: intraperitoneal injection of 0.9% NaCl (equivalent to 0 mg/kg CdCl₂); CdCl₂ treatment groups: mice received intraperitoneal injections of CdCl₂ at doses of 1.0, 2.5, or 5 mg/kg body weight once daily for 7 consecutive days. NAC pretreatment group: mice received NAC (400 mg/kg body weight, i.p.) once daily, followed 2 h later by CdCl₂ (5 mg/kg body weight, i.p.), for 7 consecutive days. After the 1-week exposure period, all mice were intravenously injected with VPS (3 mg/kg body weight) and subjected to in vivo imaging analysis. This study was ethically approved by the Biology and Medical Ethics Committee of the University of South China. The mice received care in strict accordance with the guidelines established by the Committee on the Use and Care of Animals in Hunan Province, P. R. China.

**Figure S1.** Synthetic Route for Probe **VPS**.

***Chemical synthesis.*** **VPS** was synthesized according to the routes shown in Figure S1. Compounds 1 and 2 were synthesized according to literature procedures. [S1]

[S1] Gao, X.Y., Feng, G.X., Manghnani, P.N., Hu, F., Jiang, N., Liu, J.Z., Liu, B., Sun, J.Z., Tang, B.Z., 2017. A two-channel responsive fluorescent probe with AIE characteristics and its application for selective imaging of superoxide anions in living cells. *Chem Commun* **53**, 1653–1656.

*Compound* **VPN**: 4-(Dimethylamino)cinnamaldehyde (175 mg, 1 mmol) and 2-(quinolin-4-yl)acetonitrile (168 mg, 1 mmol) were added to 10 ml of ethanol, then piperidine (26 mg, 0.3 mmol) was added and the mixture was stirred at 80 ℃ for 5 h. After completion of the reaction, as monitored by TLC, the mixture was cooled to room temperature. The solvent was removed under reduced pressure, and the crude product was purified by column chromatography (CH_2_Cl_2_:PE = 9:1, v/v) to afford an orange solid. Yield: 260 mg (80 %).^1^H NMR (500 MHz, MeOD, Figure S2) *δ* 8.87 (d, J = 4.5 Hz, 1H), 8.32 (d, J = 8.5 Hz, 1H), 8.11 (d, J = 8.5 Hz, 1H), 7.85 (t, J = 7.7 Hz, 1H), 7.72 (t, J = 7.6 Hz, 1H), 7.59 (dd, J = 10.1, 7.7 Hz, 2H), 7.52 (d, J = 8.7 Hz, 2H), 7.32 – 7.18 (m, 2H), 6.78 (d, J = 8.7 Hz, 2H), 3.05 (s, 6H). ^13^C NMR (126 MHz, MeOD, Figure S3) *δ* 151.98, 149.62, 145.89, 130.10, 129.45, 129.21, 127.25, 124.42 , 120.47, 118.89, 111.79, 38.86. HRMS (Figure S4) m/z: calcd for C_22_H_19_N_3_ [M+H]^+^: 326.1657, found: 326.1661.

**Synthesis of VPS**: Compound VPN (163 mg, 0.5 mmol) and Compound 2 (193 mg, 0.5 mmol) were added to 10 ml of acetonitrile, and the mixture was heated to reflux at 90 ℃ for 2 h. After completion of the reaction, as monitored by TLC, the mixture was cooled to room temperature. The solvent was removed under reduced pressure, and the crude product was purified by column chromatography (CH₂Cl₂:CH₃OH = 30:1, v/v) to afford a blue solid. Yield: 259 mg (82 %).^1^H NMR (500 MHz, MeOD, Fig. S5) *δ* 8.73 (d, J = 8.5 Hz, 1H), 8.43 (d, J = 9.0 Hz, 1H), 8.19 (d, J = 6.3 Hz, 1H), 8.17 – 8.14 (m, 1H), 8.07 (d, J = 11.0 Hz, 1H), 8.03 – 7.99 (m, 1H), 7.90 – 7.84 (m, 6H), 7.75 (d, J = 8.7 Hz, 2H), 7.62 – 7.57 (m, 3H), 7.52 – 7.49 (m, 4H), 7.37 (d, J = 8.6 Hz, 2H), 7.29 (d, J = 8.6 Hz, 4H), 6.24 (s, 2H), 3.22 (s, 6H).^13^C NMR (126 MHz, MeOD, Fig. S6) *δ* 155.98, 152.43, 151.29, 151.23, 148.82, 148.20, 138.56, 135.67, 132.97, 132.89, 131.69, 131.48, 131.39, 130.69, 130.23, 130.16, 130.01, 129.99, 129.69, 129.26, 129.04, 128.76, 128.65, 127.39, 127.01, 122.51, 121.53, 121.49, 120.59, 119.52, 116.61, 116.07, 59.75, 53.46, 42.34. HRMS (Fig. S7) m/z: calcd for C_41_H_35_N_3_O_2_P^+^ [M]^+^: 632.2461, found: 632.2465.

**Additional Table and Figures**

**Table S1.** Summary of representative fluorescent probes.

| **Probe** | | **Chemical structure** | | **λ_ex_/λ_em_ (nm)** | | **Biomarkers** | | **Imaging organ** | | | **Application** |  |
| --- | --- | --- | --- | --- | --- | --- | --- | --- | --- | --- | --- | --- |
| **Single-analyte probes applied to liver- or kidney-specific injury imaging** | | | | | | | | | | | |  |
| 16 | |  | | 738/740 | | O₂^•⁻^ | | Liver | | | Drug-induced liver injury |  |
| 17 | |  | | 462/668 | | O₂^•⁻^ | | Kidney | | | Acute kidney injury |  |
| 18 | |  | | 600/710 | | Polarity | | Liver | | | Acute alcoholic liver injury |  |
| 19 | |  | | 450/470-570 | | Polarity | | Kidney | | | Acute kidney injury |  |
| 20 | |  | | 766/806 | | Viscosity | | Liver | | | Acute alcoholic liver injury |  |
| 21 | |  | | 726/760 | | Viscosity | | Kidney | | | Diabetic kidney injury |  |
| **Single-analyte probes applied to hepatorenal injury imaging** | | | | | | | | | | | |  |
| 22 | |  | | 560/676 | | Viscosity | | Liver  Kidney  slices | | | Liver and kidney injury |  |
| 23 | |  | | 808/972-974 | | Hg²⁺ | | Liver, Kidney | | | Hg²⁺-triggered acute liver and kidney injury |  |
| 24 | |  | | 442/500,710 | | HClO | | Liver  Kidney | | | APAP-induced liver and kidney injury |  |
| 25 | |  | | 670/710 | | H₂O₂ | | Liver  Kidney | | | Diabetic liver and kidney injury in type 2 diabetes |  |
| 26 | |  | | 490/665 | | ONOO⁻ | | Liver  Kidney | | | Drug-induced liver and kidney injury |  |
| **Multiparameter responsive probes in different biological applications** | | | | | | | | | | | |  |
| 27 | |  | | 495/645  405/525 | | Polarity/Viscosity  Esterase | | / | | | Ferroptosis-associated liver injury |  |
|  | 28 | |  | | 570/747-810  461/544 | | Polarity/Viscosity  Nitroreductases | | / | Cancer bioimaging | | |
|  | 29 | |  | | 570/714-786  400/634 | | Polarity/Viscosity  H₂S | | / | Non-alcoholic fatty liver disease | | |
|  | 30 | |  | | 550/700  405/400-800  400/600 | | Polarity  Viscosity  LAP | | / | Inflammation-cancer progression, Epithelial-mesenchymal transition | | |
|  | 31 | |  | | 570/752-792 443/593 | | Polarity/Viscosity  Carboxylesterases | | / | AA-induced liver injury | | |
|  | 32 | |  | | 448/470-494  570/658 | | Polarity/Viscosity  ONOO⁻ | | / | Cancer cell discrimination | | |
|  | 33 | |  | | 570/750  420/650 | | Polarity/Viscosity  H₂O₂ | | / | Cancer cell discrimination | | |
|  | 34 | |  | | 570/700  430/645 | | Polarity/Viscosity  O₂^•⁻^ | | Liver | Amiodarone-induced liver injury | | |
|  | **This work** | | | | | | | | | | | |
|  | This work | |  | | 600/740  480/650 | | Polarity/Viscosity  O₂^•⁻^ | | Liver  Kidney | Cd^2+^ induced liver/kidney injury | | |

**Figure S2.** ^1^H NMR spectra of VPN in MeOD.

**Figure S3.** ^13^C NMR spectra of VPN in MeOD.

**Figure S4.** HRMS spectra of VPN.

**Figure S5.** ^1^H NMR spectra of **VPS** in MeOD.

**Figure S6.** ^13^C NMR spectra of **VPS** in MeOD.

**Figure S7.** HRMS spectra of **VPS**.

**Figure S8.** HPLC chromatograms of VPS and VPN. HPLC analysis was performed using a Thermo Fisher U3000 system equipped with an Acclaim PolarAdvantage II C18 column (150 mm × 4.6 mm, 5 μm). The mobile phase was methanol/H₂O = 95:5 (v/v). Detection wavelength: 254 nm.

**Figure S9.** The absorption spectra of **VPS** (10 μM) in solvents of different polarity.

**Figure S10.** Fluorescence spectra of **VPS** (10 μM) in different solvents. λ_ex_/λ_em_ = 600/740 nm.

**Figure S11.** Fluorescence intensity of **VPS** (10 μM) at 740 nm in different polarity solutions (PBS-1,4-dioxane system, containing 0% and 90% 1,4-dioxane, respectively) under different pH. λ_ex_ = 600 nm, λ_em_ = 740 nm.

**Figure S12.** The absorption spectra of **VPS** (10 μM) in H_2_O and glycerol.

**Figure S13.** Fluorescence spectra of **VPS** in H_2_O and 90 % glycerol. λ_ex_/λ_em_ = 600/740 nm.

**Figure S14.** (A) Fluorescence spectra of VPS (10 μM) in methanol–glycerol mixtures with different glycerol fractions. (B) Linear relationship between fluorescence intensity at 740 nm and viscosity. λ_ex_ = 600 nm.

**Figure S15.** pH effect on the fluorescence intensity of **VPS** (10 μM) at 740 nm in water-glycerol mixtures with 0% glycerol and 90% glycerol. λ_ex_ = 600 nm, λ_em_ = 740 nm.

**Figure S16.** Absorption spectra of **VPS** (10 μM) without or with O₂^•⁻^ (80 μM).

**Figure S17.** Fluorescence spectra of **VPS** (10 μM) without or with O_2_^•-^ (80 μM).

**Figure S18.** The fluorescent response of **VPS** both in the absence and presence of O₂^•⁻^ (60 μM) in the pH range of 3.0 to 11.0.

**Figure S19.** Time-dependent fluorescence intensity of **VPS** (10 μM) at 650 nm in the presence of O₂^•⁻^ (60 μM).

**Figure S20.** Fluorescence response of **VPS** (10 μM) to different analytes. λ_ex_/λ_em_ = 480/650 nm. Error bars are ±SD, n = 3.

**Figure S21.** Selective competition response of VPS (10 μM) toward O₂^•⁻^ in the presence of various potentially interfering species (100 μM unless otherwise specified) 1, Blank; 2, O₂^•⁻^ (80 μM); 3, Na⁺; 4, Ca²⁺; 5, Fe³⁺; 6, Cl⁻; 7, HSO₃⁻; 8, SO₃²⁻; 9, ClO₄⁻; 10, Zn²⁺; 11, F⁻; 12, Br⁻; 13, Hcy; 14, H₂O₂; 15, ·OH; 16, ONOO⁻ (20 μM); 17, Cys; 18, SCN⁻; 19, GSH; 20, HClO.

**Figure S22.** HR-MS of the reaction mixture of **VPS** with O_2_^•-^. The peak at m/z = 326.1668 can be assigned to be compound VPN, and the peak at m/z = 632.2470 can be assigned to be the unreacted **VPS**.

**Figure S23.** (A) Optimized structures of **VPS** and VPN in the ball-and-stick model. (B) Frontier molecular orbitals of **VPS** and VPN. The data were calculated by B3LYP/6-31G(d,p) based on the optimized ground state geometries.

**Figure S24.** Fluorescence spectra of VPS (10 μM) under different conditions: VPS alone (10 μM), VPS + O₂^•⁻^ (60 μM), VPS + 50% glycerol, VPS + 50% dioxane, and VPS + 50% dioxane + 50% glycerol. (λ_ex_ = 480 nm in (A); λ_ex_ = 600 nm in (B)).

**Figure S25.** Fluorescence spectra of VPN and VPS under different polarity and viscosity conditions. From bottom to top, the spectra correspond to VPS, VPN, VPN in H₂O/1,4-dioxane (10:90, v/v), VPN in H₂O/glycerol (10:90, v/v), VPS in H₂O/glycerol (10:90, v/v), and VPS in H₂O/1,4-dioxane (10:90, v/v), respectively. λ_ex_/λ_em_ = 600/740 nm.

**Figure S26.** Cell viability of HepG2 and HK-2 cells after incubation with different concentrations of VPS (0, 2.5, 5, 7.5, 10, 15, and 20 μM) for 12 h (A) or 24 h (B), as determined by the MTT assay. Data are presented as mean ± SD (n = 3).

**Figure S27.** Colocalization images of HepG2 cells (A) and HK-2 cells (B) with VPS and commercial dyes. Cells were stained with 500 nM commercial dyes and 10 μM VPS for 30 min. Green channel for commercial dyes, λ_ex_ = 488 nm, λ_em_ = 500−550 nm. Red channel for VPS, λ_ex_ = 561 nm, λ_em_ = 700−760 nm. Scale bar: 10 μm.

**Figure S28.** (A) Polarity-responsive fluorescence images of HepG2 cells incubated with **VPS** (10 μM, 30 min) in the absence or presence of CCCP (10 or 20 μM, 30 min). (B) Viscosity-responsive fluorescence images of HepG2 cells incubated with **VPS** (10 μM, 30 min) in the absence or presence of Nys or Mon (10 or 20 μM, 30 min). (C, D) Quantification of fluorescence intensities from (A) and (B), respectively. (E) Fluorescence images of HepG2 cells for O₂^•⁻^ detection after incubation with **VPS** (10 μM), without or with PMA (2, 4, or 6 μg/mL), PMA (6 μg/mL) plus Tiron (200 μM, 1 h), TEMPO (200 μM, 1 h), or NAC (500 μM, 1 h). (F) Quantification of fluorescence intensities from (E). Fluorescence imaging of different stress models using **VPS**. Scale bar: 20 μm.

**Figure S29.** Fluorescence microscopy analysis of HK-2 cells using **VPS**. (A) Polarity-dependent fluorescence microscopy images of HK-2 cells incubated with 10 μM **VPS** for 30 min, without or with CCCP (10 μM or 20 μM) for 30 min. (B) Viscosity-dependent fluorescence microscopy images of HK-2 cells incubated with 10 μM **VPS** for 30 min, without or with Nys (10 μM, 20 μM) or Mon (10 μM, 20 μM) for 30 min. (C, D) Quantification of fluorescence intensities from images in (A) and (B). (E) Fluorescence microscopy images of HK-2 cells incubated with 10 μM **VPS** for O₂^•⁻^ detection, with or without PMA (2, 4, 6 μg/mL), PMA (6 μg/mL)+Tiron (200 μM, 1 h), PMA (6 μg/mL)+TEMPO (200 μM, 1 h), or PMA (6 μg/mL) + NAC (500 μM, 1 h). (F) Quantification of fluorescence intensities from images in (E). Fluorescence microscopy analysis under different stress models using **VPS**. Scale bar: 20 μm.

**Figure S30.** (A) Inflammation model: Fluorescence microscopy images of HK-2 cells incubated with 10 μM **VPS**, without or with LPS (4 μg/mL or 8 μg/mL for 12 h) and LPS (8 μg/mL for 12 h) + NAC (500 μM, 1 h). (B, C) Quantification of fluorescence intensities in the O₂^•⁻^ channel (B) and viscosity/polarity channels (C) from images in (A). (D) Ferroptosis model: Fluorescence microscopy images of HK-2 cells incubated with 10 μM VPS, without or with Era (10 or 20 μM, 12 h), or Era (20 μM, 12 h) plus Fer-1 (10 μM, 12 h). (E, F) Quantification of fluorescence intensities in the O₂^•⁻^ channel (E) and viscosity/polarity channels (F) from images in (D). Scale bar: 20 μm.

**Figure S31.** Flow cytometry analysis of the O₂^•⁻^ channel is shown in HepG2 and HK-2 cells in (A, B), and of the viscosity/polarity channel in (C, D).

**Figure S32.** Heatmaps of RNA-seq–identified DEGs in kidney and liver tissues are shown in (A) and (D), respectively. KEGG pathway enrichment analyses are shown in (B) and (E), respectively. The volcano plot of DEGs in kidney tissue is shown in (C). Relative Hmox1 mRNA levels in kidney and liver tissues are shown in (F) and (G), respectively.

**Figure S33.** Relative Hmox1 mRNA levels in HK-2 and HepG2 cells are shown in (A, B). Western blot analysis of GPX4, SLC7A11, and HMOX1 in HK-2 and HepG2 cells under control or CdCl₂ treatment with or without ZnPP, Fer-1, or NAC is shown in (C, D), with corresponding quantification in (E, F). Data are presented as mean ± SD. *P < 0.05, **P < 0.01, ***P < 0.001. Data are presented as mean ± SD (n = 3).

**Figure S34.** (A) CdCl₂-induced oxidative stress model in zebrafish. Fluorescence images of zebrafish larvae incubated with VPS under the following conditions: VPS alone; CdCl₂ (10 μM, 24 h) followed by VPS; CdCl₂ (20 μM, 24 h) followed by VPS; and CdCl₂ (20 μM, 24 h) plus NAC (500 μM, 1 h) followed by VPS. VPS: 10 μM, 30 min. (B, C) Quantification of fluorescence intensities in the O₂^•⁻^ channel (B) and viscosity/polarity channel (C) from (A). Data are presented as mean ± SD (n = 3). (scale bar, 100 μm)

**Figure S35.** In vivo biocompatibility of **VPS**. (A) H&E staining of major organs (heart, liver, spleen, lung, kidney) 24 h after intravenous injection of **VPS** (scale bar, 100 μm). (B) Serum ALT and AST levels after **VPS** injection. (C) Serum BUN and CRE levels after **VPS** injection. Normal mice without **VPS** served as controls (n= 3). (D) Hemolysis of red blood cells induced by **VPS** at indicated concentrations (n=3). (E) Quantification of hemolysis based on normalized absorbance.

**Figure S36.** (A–D) Serum ALT, AST, BUN, and CRE levels in control and CdCl₂-treated mice under different treatments. Data are presented as mean ± SD (n = 3 mice per group).

**Figure S37.** H&E staining of liver and kidney tissues showing histopathological changes induced by CdCl₂ exposure and the protective effect of NAC. Representative H&E images are shown (n = 3 mice per group). Scale bar: 100 μm. Arrows indicate pathological changes.

**Figure S38.** Changes in body weight of control and CdCl₂-exposed mice under different treatments. Data are presented as mean ± SD (n = 3 mice per group).

**Figure S39.** Ex vivo fluorescence imaging in a CdCl₂-induced mouse model. (A) Ex vivo fluorescence images of major organs acquired in the O₂•⁻ channel. (B) Quantification of relative O₂•⁻ fluorescence intensities from (A). (C) Ex vivo fluorescence images of major organs acquired in the viscosity/polarity channel. (D) Quantification of relative viscosity/polarity fluorescence intensities from (C). Data are presented as mean ± SD (n = 3 mice per group).

**Figure S40.** (A) Ex vivo fluorescence imaging of kidneys in O₂^•⁻^ and viscosity/polarity channels, with corresponding quantification (B, C). (D) Ex vivo fluorescence imaging of livers in O₂^•⁻^ and viscosity/polarity channels, with corresponding quantification (E, F). Data are presented as mean ± SD (n = 3 mice per group).

**Figure S41.** Fluorescence imaging of tissue slices in an acute CdCl_2_ exposure mouse model. (A) Representative fluorescence images of liver tissue slices from mice treated with different doses of CdCl_2_ or CdCl_2_ + NAC, acquired in the green channel, red channel, bright-field, and merged images. (B, C) Quantification of relative fluorescence intensities in the green (B) and red (C) channels corresponding to liver tissues in (A). (D) Representative fluorescence images of kidney tissue slices from mice treated with different doses of CdCl_2_ or CdCl_2_ + NAC, acquired in the green channel, red channel, bright-field, and merged images. (E, F) Quantification of relative fluorescence intensities in the green (E) and red (F) channels corresponding to kidney tissues in (D). Data are presented as mean ± SD (n = 3). Scale bar: 50 μm.
